# Off-Target-Based Design of Selective HIV-1 PROTEASE Inhibitors

**DOI:** 10.3390/ijms22116070

**Published:** 2021-06-04

**Authors:** Gabriele La Monica, Antonino Lauria, Alessia Bono, Annamaria Martorana

**Affiliations:** Dipartimento di Scienze e Tecnologie Biologiche, Chimiche e Farmaceutiche “STEBICEF”, Università degli Studi di Palermo, Viale delle Scienze—Ed, 17-90128 Palermo, Italy; gabriele.lamonica01@unipa.it (G.L.M.); alessia.bono01@community.unipa.it (A.B.); annamaria.martorana@unipa.it (A.M.)

**Keywords:** molecular docking, molecular descriptors, ligand-structure based, DRUDIT, on/off-targets, virtual screening, HIV-1 protease, NCI database

## Abstract

The approval of the first HIV-1 protease inhibitors (HIV-1 PRIs) marked a fundamental step in the control of AIDS, and this class of agents still represents the mainstay therapy for this illness. Despite the undisputed benefits, the necessary lifelong treatment led to numerous severe side-effects (metabolic syndrome, hepatotoxicity, diabetes, etc.). The HIV-1 PRIs are capable of interacting with “secondary” targets (off-targets) characterized by different biological activities from that of HIV-1 protease. In this scenario, the in-silico techniques undoubtedly contributed to the design of new small molecules with well-fitting selectivity against the main target, analyzing possible undesirable interactions that are already in the early stages of the research process. The present work is focused on a new mixed-hierarchical, ligand-structure-based protocol, which is centered on an on/off-target approach, to identify the new selective inhibitors of HIV-1 PR. The use of the well-established, ligand-based tools available in the DRUDIT web platform, in combination with a conventional, structure-based molecular docking process, permitted to fast screen a large database of active molecules and to select a set of structure with optimal on/off-target profiles. Therefore, the method exposed herein, could represent a reliable help in the research of new selective targeted small molecules, permitting to design new agents without undesirable interactions.

## 1. Introduction

### 1.1. Proteases: Key Enzymes for HIV Maturation

Retroviral HIV-1 PRotease (HIV-1 PR), also called retropepsin, plays an essential role in the process of maturation of non-infectious spherical virions. Similar to other proteases, in specific proteolytic cleavage sites, HIV-1 PR processes polyproteins (especially *Gag* and *Gag-Pol*, associated with virion membrane) to yield functional individual subunits (structural proteins such as MA, CA, and NC that are involved in the stabilization of the lipidic envelope, capsid, and nucleocapsid, respectively; and enzymes such as reverse transcriptase, protease, and integrase) [1,2]. The inefficient or defective activity of the protease leads to unmatured viral particles with reduced/absent infectivity [3,4,5]; for this reason, HIV-1 PR has long been studied and represents, even today, a key target for AIDS therapy.

From a biomolecular point of view, HIV-1 PR is a small aspartic protease, which is formed in its active form, by two identical monomers of 99 amino acids interacting with each other at a dimer-interface, through a β-sheet structure. Each monomer folds into a compact structure of β-strands with a short α-helix near the C terminus. The pseudo-symmetric active site appears to be a central cavity, equally defined at the interface of the two monomers by residues 8, 23–32, 45–56, 76, and 80–84 from both subunits; each of them exposes the highly conserved catalytic triads Asp^25^, Thr^26^, and Gly^27^, involved in the mechanism of action [1,6,7,8,9,10,11,12].

The mutational analysis of both Asp^25^ residues demonstrates their central role in the catalysis. The substitutions with Asn, Thr, or Ala lead to an enzyme without any proteolytic activity [5,9,13,14,15]. These two key residues are planar and interact strictly with substrates and inhibitors [16]. During the catalytic mechanism, these residues coordinate a molecule of water to hydrolyze the specific peptide bond, with the formation of an oxyanion tetrahedral intermediate (general acid-base catalysis) [15,17,18,19].

A flexible glycine-rich “flap” region (residues 44–57) consisting of two β-hairpins, covers the active cleft, and cooperates in substrate recognition and stabilization and in the regulation of the catalytic activity. Indeed, depending on the binding of a substrate or of a small molecule, this region undergoes conformational changes, assuming an open, a semi-open, or a close conformation [20,21,22]. The endogenous substrates present at least seven amino acids and a cleavable peptide bond located between the fourth and fifth residues, starting from the N terminus [23].

### 1.2. Inhibitors of HIV Protease: Main Features and off-Target Effects

**Figure 1:**
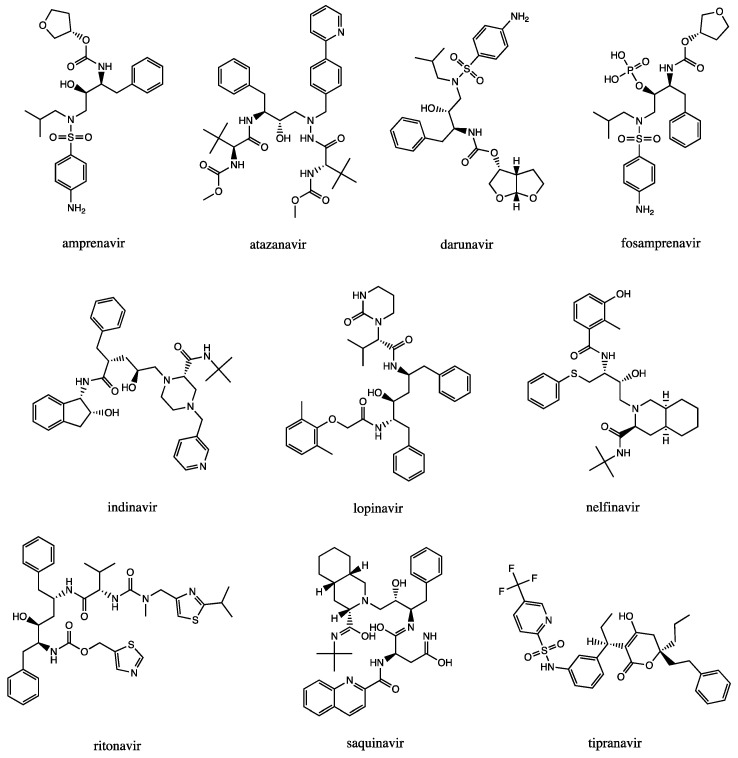
Chemical structures of the HIV-1 PR inhibitors approved by the FDA since 1995.

The introduction in clinical practice of saquinavir (1995) (Figure 1)*,* the first HIV-1 PRI (HIV-1 PRotease Inhibitor), represented an important step for AIDS therapy. Indeed, even today, the combination therapy of HIV-1 PRI and reverse transcriptase inhibitors (HAART, highly active antiretroviral therapy) is the standard pharmaceutical approach [24,25].

**Figure 2:**
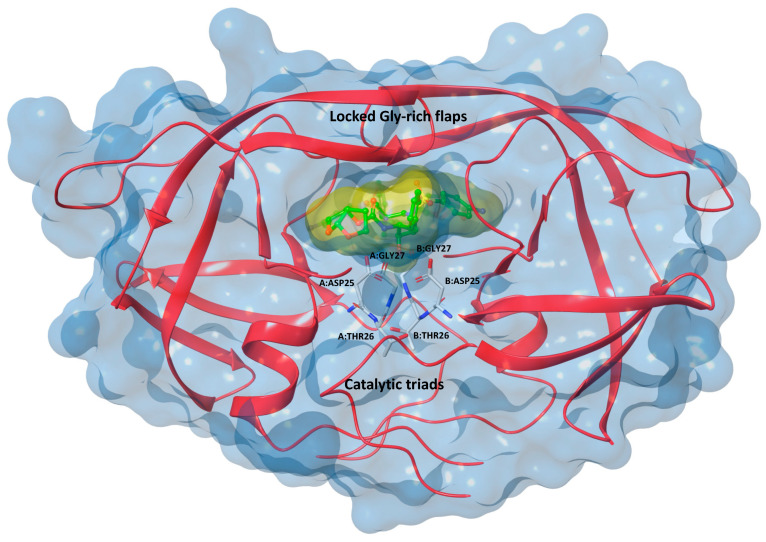
3D structure of HIV-1 PR in complex with darunavir (PDB id: 2IEN), with the two catalytic triads (Asp^25^, Thr^26^, and Gly^27^) highlighted [26].

All FDA-approved HIV-1 PRIs (saquinavir and amprenavir are no longer marketed; ritonavir, fosamprenavir, lopinavir, indinavir, atazanavir, nelfinavir, tipranavir and darunavir used for clinical treatment, Figure 1) are competitive inhibitors at the catalytic active site, binding the protease in its closed conformation (flaps folded over the active site, as in the crystal structure of HIV-1 protease in complex with darunavir, PDB id: 2IEN, Figure 2). Through this mechanism of action, the inhibitors maintain the enzyme in a locked-down state, hindering access to natural substrates [24,26,27].

Generally, from a structural point of view, the central moieties of HIV-1 PRIs present a set of non-hydrolyzable hydrocarburic chains (hydroxyethylene, hydroxyethyl amine, and hydroxyethylenamino-sulfonamide), which mimic the tetrahedral-hydroxy catalytic intermediates of the peptide substrates and form favorable electrostatic interactions with the two pivotal Asp^25^ amino acids [15,28].

Besides the increasing drug-resistance to conventional therapy [10,11,29], another important problem in the treatment of HIV/AIDS patients with HIV-1 PRIs is the variety of the targets, which, as “minor” targets, could be activated after HIV-1 PRIs administrations [30]. Drug promiscuity represents the molecular basis for polypharmacology [31], that is, the capability of a compound to interact with multiple targets. In some multifactorial diseases, such as cancer or Alzheimer, the interaction with multiple interconnected targets can be advantageous. In other cases, this promiscuity leads to interaction with not desired/harmful targets (called off-targets or anti-targets) that are frequently responsible for mediating side effects [32].

The harmful promiscuity of HIV-1 PRIs is well-known. Several studies demonstrated the capability of many PRIs to inhibit not only the primary target, but also other ones involved in the regulation of glucose and lipids homeostasis, as well as cell proliferation and survival [30,33,34,35,36]. Indeed, from a biomolecular point of view, this evidence is reported in the literature and describes the capability of HIV-1 PRIs to inhibit the Akt, EGFR, and IGF1-R pathways involved in the regulation of metabolic processes, and also investigated in anticancer therapy [35,36,37,38,39,40,41,42].

The interactions of HIV-1 PRIs with the off-targets are in part the cause of the often-unbearable side effects—dyslipidemia, hepatotoxicity, insulin resistance, diabetes, and lipodystrophy/lipoatrophy, in addition to cardiovascular and cerebrovascular diseases. The off-target effects represent an important drawback, especially in the light of lifelong treatment for patients infected by HIV. Indeed, despite the undisputed benefits, these side effects negatively affect their quality of life [1,34,37,43,44].

According to Lv et al., the use of new scaffolds or lead compounds in the design of HIV protease inhibitors might lead to alternative binding patterns, becoming a possible solution to the reduction of drug side effects of the FDA-approved PRIs [30]. Therefore, the prediction of off-target interactions, through polypharmacological computational approaches, already in the early stages of drug discovery and development process, could strongly help pharmaceutical research, saving time and resources, and reducing the risk of failure [32,45,46,47]. In this regard, Xie et al. developed a computational bioinformatics approach that allowed us to better understand the side effects of nelfinavir, through the analysis of its putative off-targets [35,48].

Therefore, in this work, an innovative on/off-target based in silico protocol is reported, with the aim of identifying new and more selective HIV-1 PRIs. Particular attention has been paid to the analysis of the low affinity of the studied HIV-1 PRIs against the off-targets responsible for the side effects, in patients treated with approved PRIs. The protocol develops in two steps (ligand- and structure-based). The use of the ligand-based method in the first step allows us to perform fast and reliable virtual screenings, without the need for high-performance hardware and software.

## 2. Results

### 2.1. In Silico Ligand-Based Approach in the Identification of Selective HIV-1 PR Inhibitors

**Figure 3:**
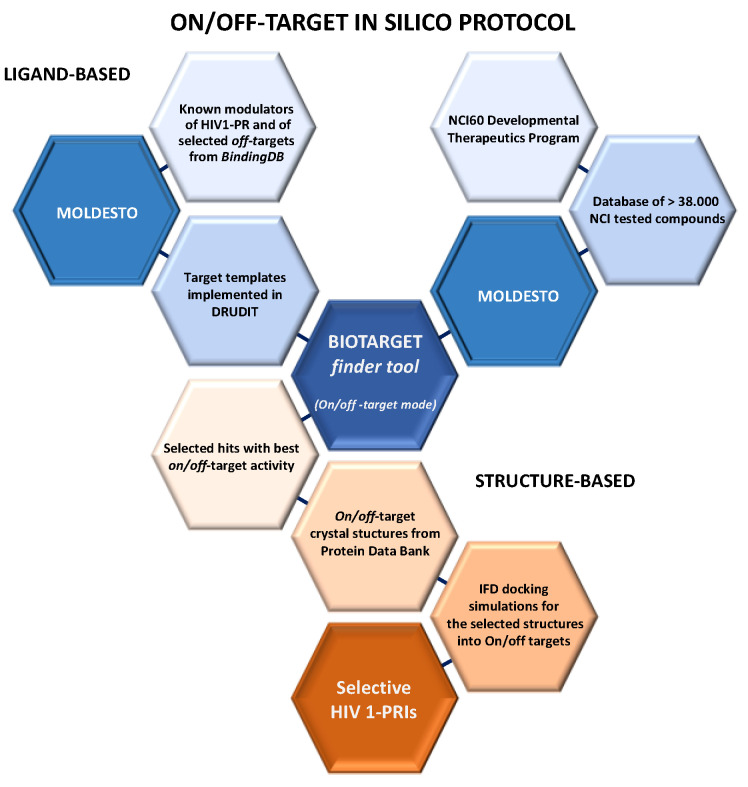
Workflow of the in silico mixed structure-ligand on/off-target approach proposed for the identification of new HIV-1 PR inhibitors.

In the first step of the protocol (Figure 3), the well-established ligand-based computational approaches centered on molecular descriptors, recently implemented in the web-service DRUDIT [49], was applied. In particular, it has been employed the BIOTARGET finder tool in an on/off-target mode (explained in the Materials and Methods section) [49].

For the building of the on/off-target templates, four ligand datasets were selected—one included the known modulators of the on-target (HIV-1 PR) and the other constituted the known inhibitors of the well-known off-targets (AKT, EGFR, and IGF1R). The BindingDB, a web-accessible database where the K_i_, K_d_, IC_50_, and EC_50_ values for thousand active molecules against the corresponding known target/s are available [50], was used as a reliable source for the modulators. These structures databases were subjected to a laborious cleaning work, with the aim of deleting duplicates and selecting only the most active compounds, fixing the IC_50_ cut-off at 1 μM. Then, the selected structures were employed to build the molecular descriptor-based target templates, according to the procedure reported in the literature [49,51]. In brief, the four ligand datasets were processed by MOLDESTO (MOLecular DEScriptors TOols, a proprietary software implemented in DRUDIT), which can calculate more than 1400 molecular descriptors (3D, 2D, and 1D) for the input structures. The output matrices (structures versus molecular descriptors), one for each target (HIV-1 PR, AKT, EGFR, and IGF1R), were converted into a sequence of a pair of values for each molecular descriptor—mean and standard deviation. The four sequences of these couple of values represented the ligand-based target templates.

Once the targets templates have been built and integrated in DRUDIT, the second step of the ligand-based study was focused on the virtual screening of a large ligand database, by means of the BIOTARGET finder tool available in DRUDIT, applying the on/off-target task. The aim of this part of the protocol was to filter a large database of structures through the templates, in order to select new compounds as potential selective HIV-1 PRIs, with low affinity against the putative off-targets responsible for mediating the side-effects, in patients treated with the approved HIV-1 PRIs.

The National Cancer Institute (NCI) molecular databank, constituted by thousands of compounds tested in the National Cancer Institute anticancer screening program (NCI60), was selected as a large ligand database to be screened [52,53]. Indeed, from a polypharmacological point of view, old, or unsuccessful lead compounds/drugs could be repurposed for new biological targets that are different from those for which they were developed; in silico approaches are also quite suitable for this purpose [31,54].

Therefore, more than 38,000 structures were submitted to the DRUDIT templates calculation. The analysis of the output matrix (Supplementary Material S1) allowed us to select 330 compounds, with Drudit Affinity Scores (DAS) higher than 0.7, against the on-target HIV-1 PR. Then, these selected structures (330) were further screened against the off-target receptors (EGFR, IGF1R, and ALK), in order to classify those with the best on/off-target balance, by applying the formula:

$$\mathrm{On}/\mathrm{off}\;\mathrm{target}\;\mathrm{score}=\frac{DAS_{HIV-1\;PR}}{DAS_{\chi }}$$

where *DAS_HIV-1 PR_* is the score for the HIV-1 PR; *DASχ* is *DAS_EGFR_* × *DAS_IGF1R_* × *DAS_ALK_*.

The selected chemical structures were ranked according to the following criterion—the higher on/off-target score value was the result of a higher *DAS _HIV-1 PR_*, with a higher affinity for the on-target HIV-1 PR, and a lower *DAS_EGFR_*, *DAS_IGF1R_*, and *DAS_ALK_* for the off-targets (Supplementary Material S1). In view of this, the first twenty best ranked structures were selected to conduct further analysis.

Moreover, to better evaluate the rankings of the selected NCI structures, the FDA-approved HIV-1 PR inhibitors (Figure 1) were also screened; the overall results are reported in Table 1. By observing the results, it emerged that 5 out of 10 approved HIV-1 PRI (ritonavir, darunavir, indinavir, nelfinavir, amprenavir) reported an on/off-target score lower than the selected NCI structures. Among all, attention should be paid to nelfinavir, the second-last ranked structure, which reported an on/off-target score of 13,575, which was much lower than the average value of the structures selected by the protocol (23,612). This was probably due to its non-negligible EGFR, IGF1R, and ALK inhibition activities that is extensively documented in the literature, explaining the metabolic and anticancer properties of this molecule [55], which, in this context, could be described to be a side-effect of HIV therapy. As expected, the lower on/off-target score reflects the evidence of considerable activity on the off-targets—EGFR, IGF1R, and ALK.

On the other hand, the other FDA-approved HIV-1 PRI were ranked within the NCI compounds; however, three of these, atazanavir, tipranavir, and fosamprenavir, obtained a DAS_HIV-1 PR_ lower than the cut-off 0.7, according to the proposed model, thus they could be excluded, according to the analysis. Thus, excluding saquinavir, which is no longer marketed, only lopinavir obtained a DAS_HIV-1_ and an on/off target score comparable to the selected NCI structures.

In the light of these results, we decided to further investigate the inhibition effects of the twenty best-scored NCI molecules, with detailed structure-based studies.

**Figure 4:**
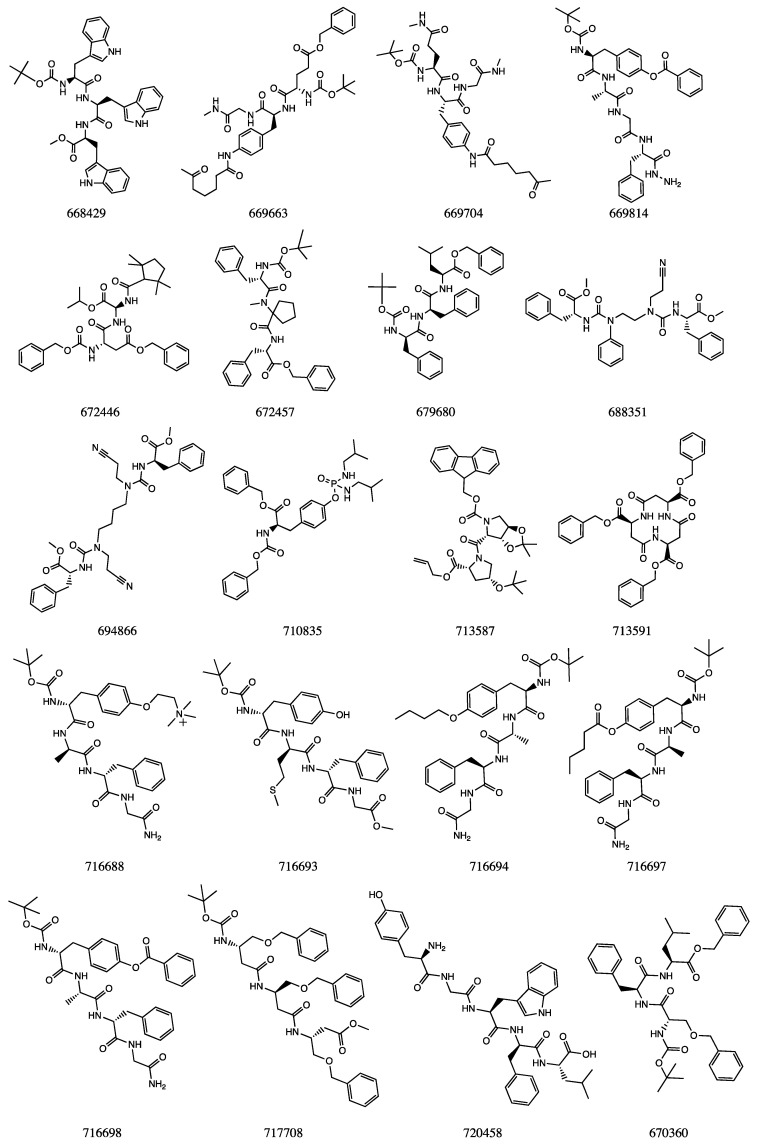
Chemical structures of the top-scored NCI compounds obtained by the ligand-based analysis (the number reported below each structure represents the NSC number in the NCI database).

In Figure 4, the twenty selected structures were reported from the analysis of their chemical structures. Most of these compounds presents a peptidomimetic and carbamic moieties, such as several already approved HIV-1 PR inhibitors.

### 2.2. In Silico Structure-Based: Molecular Docking for the Best-Scored Structures

Hybrid and hierarchical virtual screenings—composed of both sequential ligand and structure-based methods—were demonstrated to be reliable approaches in small molecule drug discovery [56]. In the second part of the protocol (Figure 3), Induced Fit Docking (IFD) simulations were processed both in on-target (HIV-1 PR, PDB id: 1HVR [57]) and off-target crystal structures (PDB id 6MX8 [58], 3W2S [59], and 5FXS [60] for ALK, EGFR, and IGF1R, respectively) selected from the Protein Data Bank [61]. The structure-based studies aimed to confirm the DRUDIT predictions and select the molecules that emerged as the most interesting (with the best on/off-target activity).

The IFD scores were analyzed and processed as follows. For each structure, the difference between the on-target docking score and the off-target docking scores was calculated. Then, the average value (Y) of these was calculated and we decided to consider only those structure with absolute Y value greater than 3.5 units (implying more favorable interactions of the ligand with the on-target instead of with the off-targets, see Supplementary Material S2). In Table 2, the docking outputs were reported for the six structures beyond the cut-off of 3.5. From the on-target point-of-view, all selected NCI molecules showed a higher IFD score against the HIV-1 PR, as compared to the co-crystallized ligand, confirming a good-fit in the catalytic binding site and improved selective interactions with the HIV-1 PR. Regarding the off-target affinity, in general, the majority of the studied molecules presented lower docking/IFD scores against the off-targets, as compared to the corresponding co-crystallized ligands, suggesting a lower affinity in accordance with the DRUDIT ligand-based results.

The matching of the IFD results with those obtained by the DRUDIT ligand-based analysis highlights the NSC672457 and NSC669704 molecules as the most promising HIV-1 PRI. Corresponding to the first and the fourth structures in the DRUDIT ranking output file, these two compounds exhibited the best IFD on-target scores against HIV-1 PR and the lower IFD off-target scores against ALK, EGFR, and IGF1R.

In this light, the binding mode and the ligand–protein interactions of the best-docked pose of NSC672457 and NSC669704 into HIV-1 PR catalytic binding site were further analyzed.

**Figure 5:**
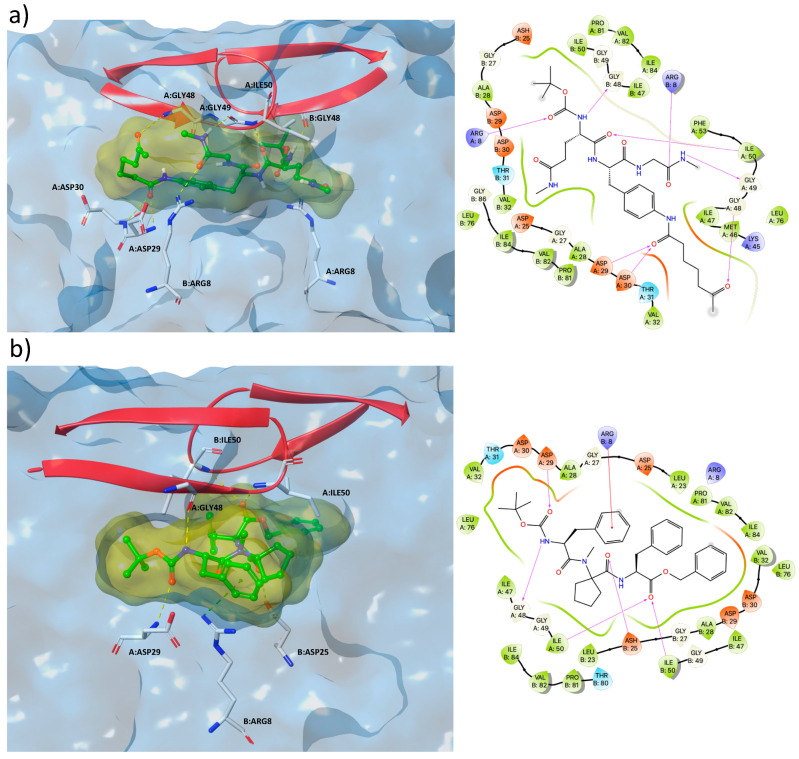
3D binding modes (left) and amino acids maps (right) of the best docked pose of NSC669704 (**a**) and NSC672457 (**b**), bound to the catalytic domain of HIV-1 PR (PDB id: 1HVR).

As depicted in Figure 5, both selected molecules bind to the HIV-1 PR catalytic domain in an extended conformation, in accordance with the binding mode generally reported in the literature for other protease inhibitors [29]. Furthermore, NSC672457 and NSC669704 compounds interact with pivotal amino acids in the proteolytic active site with ASPs^25^, ASPs^29^, and ASPs^30^. H-bonds with Ile^50^, Gly^48^, and Gly^49^, within the two β-hairpins, could contribute to reinforcing the inhibition effect by locking the flaps (highlighted as red ribbons) in a closed conformation and hindering the entrance of the natural peptide substrates.

Interestingly, NSC669704 appears for the first time in a virtual screening as putative HIV-1 PRI, while NSC672457 has been identified as a possible HIV-1 PRI, through a hierarchical virtual screening [62]. This reinforces the idea that the integration of our ligand-based tools with conventional structure-based techniques represents a valid and reliable method to screen a wide library of compounds in the targeted drug discovery field.

## 3. Materials and Methods

### 3.1. Ligand-Based Studies

The selection of suitable HIV-1 PR inhibitors was performed through the BIOTARGET finder on/off module, available in the DRugs Discovery Tools (DRUDIT) web-service (www.drudit.com) (accessed on 1 May 2021) [49]. The tool allowed analysis of the binding affinity of the candidate molecules versus a chosen biological on-target, also taking into account the correspondence to the related off-targets.

In detail, as a first step, it was necessary to build the DRUDIT templates of the biological targets of interest (on-target—HIV-1 PR; off-targets—AKT, EGFR, and IGF1R), using a set of well-known selective inhibitors as the reference compounds. Therefore, from the Binding DataBase [50], a wide cluster of molecules were downloaded and further filtered, by applying a cut-off of 1 μM to their IC_50_ values. The selected structures were uploaded to the web-server DRUDIT and processed by the MOLecular DEScriptor TOol (MOLDESTO), which can deal with more than one thousand and four hundred molecular descriptors.

MOLDESTO can read common molecule file formats, such as SMILES, SDF, Inchi, Mdl, and Mol2, to optimize structures, and is provided with a caching system to boost the calculation speed of previously submitted structures. Input structures can be drawn in the web application or uploaded to the server as external files. In either case, the structures were optimized by MOPAC before being processed by MOLDESTO. The consequent output files allowed us to obtain the expected DRUDIT biotarget templates of HIV-1 PR, AKT, EGFR, and IGF1R, which are necessary for the on/off-target screening, and in this regard, added to the list of the biological targets already available in the DRUDIT platform.

In the second step of the work, the NCI database, characterized by well-known antiproliferative data, was screened and chosen as a molecule source, to be uploaded to DRUDIT. In this phase of the workflow, the BIOTARGET finder Tool (including the new biological templates of HIV-1 PR, AKT, EGFR, and IGF1R) was selected, using the default parameters, at first, and then further analyzing the output files by ticking the on/off option.

### 3.2. Structure-Based Studies

The ligands and protein–ligand complexes used for the in silico studies were prepared as follows.

#### 3.2.1. Ligand Preparation

The default setting of the LigPrep tool implemented in Schrödinger’s software (version 2017-1) was used to prepare the ligands [63]. All possible tautomers and a combination of stereoisomers were generated for a pH of 7.0  ±  0.4, using the Epik ionization method [64]. Energy minimization was subsequently carried out using the integrated OPLS 2005 force field [65].

#### 3.2.2. Protein Preparation

The high-resolution crystal structure of HIV-1 PR, (PDB id: 1HVR [57]) and off-target crystal structures of ALK (PDB id 6MX8 [58]), EGFR (3W2S [59]), and IGF1R (5FXS [60]) were downloaded from the Protein Databank [61,66]. The Protein Preparation Wizard of the Schrödinger software was subsequently employed for further preparations of the protein structures using the default setting [67]. Bond orders were assigned and hydrogen atoms were added, as well as protonation of the heteroatom states were carried out using the Epik-tool (with the pH set at biologically relevant values, i.e., at 7.0  ±  0.4). The H-bond network was then optimized. The structure was finally subjected to a restrained energy minimization step (RMSD of the atom displacement for terminating the minimization was 0.3 Å), using the Optimized Potentials for Liquid Simulations (OPLS) 2005 force field [65].

#### 3.2.3. Docking Validation

Molecular Docking studies were performed by the Glide program [68,69,70]. The receptor grids preparation was carried out by assigning the original ligands as the centroid of the grid box. The generated 3D conformers were docked into the receptor model using the Standard Precision (SP) mode as the scoring function. A total of 5 poses per ligand conformer were included in the post-docking minimization step, and a maximum of 2 docking poses were generated for each ligand conformer. The proposed docking procedure was able to re-dock the original ligands within the receptor-binding pockets with RMSD  <  0.51 Å.

#### 3.2.4. Induced Fit Docking

Induced fit docking simulation was performed using the IFD application [71,72] available in the Schrödinger software suite [73], which was demonstrated to be an accurate and robust method to account for both ligand and receptor flexibility [74]. The IFD protocol was carried out as follows [75,76]—the ligands were docked into the rigid receptor models with scaled-down van der Waals (vdW) radii. The Glide Extra Precision (XP) mode [68,69,70] was used for the docking, and 20 ligand poses were retained for the protein structural refinements. The docking boxes are defined to include all amino acid residues within the dimensions of 25 Å × 25 Å × 25 Å from the center of the original ligands; the induced-fit protein–ligand complexes are generated using the Prime software [77,78]. The 20 structures from the previous step were submitted to sidechain and backbone refinements. All residues with at least one atom located within 5.0 Å of each corresponding ligand pose were included in the refinement by Prime. All poses generated were then hierarchically classified, refined, and further minimized into the active site grid before being finally scored using the proprietary GlideScore function, defined as GScore = 0.065 * vdW + 030 * Coul + Lipo + Hbond + Metal + BuryP + RotB + Site, where: vdW is the van der Waals energy term, Coul is the Coulomb energy, Lipo is a Lipophilic contact term that rewards favorable hydrophobic interactions, Hbond is an H-bonding term, Metal is a metal-binding term (where applicable), BuryP is a penalty term applied to buried polar groups, RotB is a penalty for freezing rotatable bonds, and Site is a term used to describe favorable polar interactions in the active site.

Finally, the IFD score (IFD score = 1.0 Glide_Gscore + 0.05 Prime_Energy), which accounts for both protein–ligand interaction energy and total energy of the system, was calculated and used to rank the IFD poses. The more negative was the IFDscore, the more favorable was the binding.

## 4. Conclusions

Despite the numerous efforts of the last 20 years, the therapy of HIV infection still represents a challenge, for many factors. Even today, HIV-1 PR inhibitors characterize, together with the inhibitors of reverse transcriptase, the mainstays of HIV pharmacological therapy.

If the advent of saquinavir—the first HIV-1 PR inhibitor—in 1995, symbolized a fundamental step for the treatment of HIV-infected patients, the daily and prolonged use of these agents caused many side-effects, some of which were unbearable. This is due to the capability of a lot of these drugs to interact, not only with the principal target HIV-1 PR, but also with other “secondary” targets involved in metabolic regulation and cell proliferation.

Many efforts were made along the years to select new possible inhibitors, in order to overcome the drawbacks of these class of pharmaceuticals, and in this light, computational approaches permit to dramatically reduce the time and costs of research.

In this work, we reported a new, reliable, mixed in-silico ligand/structure-based protocol that use on/off-targets, in order to select new possible leads, such as HIV-1 PR with optimal on/off-target profiles. Through our recently developed ligand-based tool available in DRUDIT (BIOTARGET finder tool), it was possible to rapidly screen and skim a large structure database (more than 38.000 compounds), selecting only those with the best activity against HIV-1 PR on-target, without a considerable affinity against the selected off-targets. The 20 best-scored molecules were further analyzed with conventionally induced fit docking protocols, in order to integrate and confirm the results obtained in the first part of the protocol. By merging the ligand and the structure-based data for the twenty selected molecules, the NSC669704 and NSC672457 molecules resulted in the best structures with optimal predicted on/off-target balance, in both ligand and structure-based studies.

The analysis of the best-docked poses of the two ligands, in complex with HIV-1 PR, showed the capability of both compounds to form interactions with the key amino acid residues within the active site, maintaining the two flap regions in a locked state over the catalytic cleft.

In summary, the proposed new in silico protocol based on on/off-targets could represent an important help in the design of new targeted agents, without unbearable promiscuous interactions. Obviously, further studies are necessary to confirm, in wet, the reliability of the protocol.

## Figures and Tables

**Table 1:** DAS data and the on/off-target scores for the twenty top-scored structures and the 10 FDA-approved HIV-1 PR inhibitors.

Cpd *	*DAS_HIV-1 PR_*	*DAS_ALK_*	*DAS_EGFR_*	*DAS_IGF1R_*	*DAS* *χ*	On/Off-Target Score
**atazanavir**	0,624	0,272	0,304	0,246	0,02034	30,677
669704	0,742	0,280	0,314	0,302	0,02655	27,945
713591	0,700	0,288	0,312	0,280	0,02516	27,822
669814	0,828	0,286	0,360	0,316	0,03254	25,449
672457	0,856	0,294	0,344	0,334	0,03378	25,341
720458	0,740	0,282	0,352	0,296	0,02938	25,185
716698	0,842	0,288	0,352	0,330	0,03345	25,169
**lopinavir**	0,816	0,332	0,332	0,316	0,03483	23,428
694866	0,736	0,284	0,346	0,322	0,03164	23,261
670360	0,852	0,314	0,358	0,326	0,03665	23,249
**saquinavir**	0,802	0,304	0,364	0,312	0,03452	23,230
716693	0,824	0,306	0,352	0,330	0,03554	23,182
672446	0,770	0,290	0,342	0,336	0,03332	23,106
716688	0,764	0,312	0,348	0,306	0,03322	22,995
679680	0,872	0,316	0,362	0,334	0,03821	22,823
713587	0,734	0,326	0,310	0,322	0,03254	22,556
716697	0,802	0,324	0,350	0,314	0,03561	22,523
668429	0,786	0,274	0,362	0,354	0,03511	22,385
669663	0,754	0,292	0,358	0,324	0,03387	22,262
**tipranavir**	0,616	0,294	0,304	0,312	0,02789	22,090
**fosamprenavir**	0,694	0,290	0,312	0,348	0,03149	22,041
688351	0,806	0,304	0,342	0,352	0,0366	22,024
717708	0,714	0,310	0,340	0,312	0,03288	21,712
716694	0,816	0,316	0,372	0,320	0,03762	21,693
710835	0,772	0,306	0,344	0,340	0,03579	21,570
**ritonavir**	0,802	0,326	0,344	0,342	0,03835	20,911
**darunavir**	0,756	0,338	0,336	0,388	0,04406	17,157
**indinavir**	0,822	0,372	0,368	0,362	0,04956	16,587
**nelfinavir**	0,908	0,404	0,416	0,398	0,06689	13,575
**amprenavir**	0,738	0,412	0,374	0,492	0,07581	9735

* in bold, the FDA-approved HIV-1 PRI are highlighted.

**Table 2:** Docking and IFD scores of the structures that emerged from the structure-based studies, with the best on/off-target activity balance.

	1HVR (HIV-1 PR)		6MX8 (ALK)		3W2S (EGFR)		5FXS (IGF1-R)	
Structures *	Docking Score	IFD Score	Docking Score	IFD Score	Docking Score	IFD Score	Docking Score	IFD Score
**672457**	−13,243	−430,376	−7201	−605,292	−12,100	−6754	−5580	−383,639
716697	−15,061	−439,048	−10,402	−615,334	−10,134	−679,858	−10,843	−395,718
**669704**	−13,491	−438,606	−8894	−614,762	−10,387	−680,854	−8810	−393,550
688351	−12,472	−431,583	−7793	−608,720	−9690	−673,572	−7832	−387,064
713587	−12,140	−430,773	−7369	−606,621	−9403	−675,306	−7750	−386,826
717708	−13,491	−434,575	−9435	−610,702	−11,361	−678,017	−8512	−386,593
Co-crystallized ligands	−14,942	−430,230	−9205	−612,33	−11,494	−678,360	−11,308	−394,020

* in bold, the structures with best on/off-target activity in both ligand and structure-based analysis are reported.

## Data Availability

Not applicable.

## Supplementary Materials

The following are available online at https://www.mdpi.com/article/10.3390/ijms22116070/s1.
